# Rapeseed and Palm Oils Can Improve the Growth, Muscle Texture, Fatty Acids and Volatiles of Marine Teleost Golden Pompano Fed Low Fish Oil Diets

**DOI:** 10.3390/foods14050788

**Published:** 2025-02-25

**Authors:** Fang Chen, Yunkun Lou, Junfeng Guan, Xue Lan, Zeliang Su, Chao Xu, Yuanyou Li, Dizhi Xie

**Affiliations:** College of Marine Sciences, South China Agricultural University, Guangzhou 510642, China; chenfang@scau.edu.cn (F.C.); z2564233004@163.com (Y.L.); a374168380@163.com (J.G.); 19812141680@163.com (X.L.); zeliangsu@stu.scau.edu.cn (Z.S.); xuc1213@scau.edu.cn (C.X.); yyli16@scau.edu.cn (Y.L.)

**Keywords:** *Trachinotus ovatus*, growth, muscle quality, fatty acid profiles, volatile compounds

## Abstract

This study evaluated the effects of different lipid sources—fish oil (FO), soybean oil, rapeseed oil, and palm oil—on the growth and muscle quality of golden pompano (*Trachinotus ovatus*) cultured in offshore cages for 10 weeks. Three diets (D1–D3) were formulated: D1 used only fish oil, D2 blended fish, rapeseed oil, and palm oil, and D3 combined fish and soybean oils. Fish in the D1 group showed the highest weight gain, specific growth rate, and muscle protein content, significantly outperforming D3. No significant differences in muscle lipid content or edible quality were found between groups. D1 had the highest levels of long-chain and n-3 polyunsaturated fatty acids (PUFA), while D3 had higher n-6 PUFA. Saturated and monounsaturated fatty acids were higher in D1 and D2 than in D3. Muscle volatiles like aldehydes and amines were elevated in D1, with more pleasant flavors compared to D2 and D3. Muscle texture was superior in D2. These results suggest that rapeseed and palm oils can enhance growth, flavor, and texture in fish on low FO diets, offering a sustainable alternative to reduce reliance on marine-based feed in aquaculture.

## 1. Introduction

Lipids, providing efficient energy and essential fatty acids (EFA), are essential nutrients for fish growth, reproduction, and maintenance [1]. Fish oil (FO), vegetable oil, and rendered animal fat are the main dietary lipid sources for aquatic feeds. Compared to vegetable oil and rendered animal fats, FO, a preferred lipid source for fish, contains a well-balanced fatty acid composition with a high content of n-3, long-chain, polyunsaturated fatty acids (n-3 LC-PUFA), for instance, eicosapentaenoic (EPA) and docosahexaenoic acids (DHA), are EFA for the growth and development of all vertebrates [2,3]. Although beneficial to fish growth, health, and nutrition, the shortage and high price of FO have resulted in the increased utilization of vegetable oil and rendered animal fats [2,4]. Increasingly, studies imply that the substitution of dietary FO with alternative lipid sources has a less significant impact on the growth performance of fish. In contrast, the substitution has a much more pronounced effect on the muscle quality of fish, particularly on the reduction of n-3 LC-PUFA content [5,6].

High muscle quality is not only limited to nutrients but also correlated to sensory experience for consumers, such as texture and flavor. Regarding sensory quality, lipid and fatty acid nutritional strategies have been conducted to manipulate the muscle texture of fish [7], while the effects of dietary lipids and fatty acids on the fish flavor have been of little concern [8]. Volatile compounds, including aldehydes, ketones, hydrocarbons, etc., are an important contributor to the flavor of fish, which determines its acceptability for consumers [9,10]. Lipids and fatty acids are the important precursors of volatile compounds through lipolysis and oxidation, which are correlated to the unsaturated bonds of fatty acids. Unsaturated fatty acids (UFA) are much more prone to lipid peroxidation and production of flavor compounds compared to saturated fatty acids (SFA) [10]. Dietary FO can result in the fish’s edible portion possessing a more pronounced odor, potentially elevating the risk of undesirable odors in the muscle to a certain extent [8]. Thus, from the view of lipids, fatty acids, and precise nutrition, it is important to balance the growth, nutrition, and flavor of cultured fish.

Golden pompano (*Trachinotus ovatus*), an increasingly popular marine fish species, is well suited to various farming modes, and its annual output has been more than 240 thousand tons in the recent two years in China [11]. Based on growth performance, fatty acid composition, health status, and gut microbiota, numerous studies have assessed the individual or combined soybean, coconut, palm, oil-tea camellia seed, olive, canola, peanut, linseed, perilla, and lard oils as dietary FO alternatives in *T. ovatus* [12,13,14,15,16]. However, the impact of the FO substitutions on the flavor of *T. ovatus* remains unclear. Therefore, the current study investigates how dietary lipid sources affect the muscle flavor quality of *T. ovatus*. Our findings will provide a basis for improving the muscle quality of farmed *T. ovatus* through dietary lipid nutrition strategies.

## 2. Materials and Methods

### 2.1. Diet Formulation

Using fish, rapeseed, and soybean oils as lipid sources, three isoproteic (~49.0%) and isolipidic (~12.0%) diets were formulated, namely, diets D1–D3. Diet D1 contained 8% fish oil; diet D2 contained an 8% mixture of fish, rapeseed, and palm oils in a ratio of 4:3:3; and diet D3 incorporated an 8% mixture of fish and soybean oils in a ratio of 2:3. The detailed feed formulations, nutrient composition, and fatty acid profiles are shown in Table S1. The process and preparation of diets are accorded to our previous studies [15,17]. Briefly, all the feed ingredients were initially processed by a grinding machine and subsequently sieved through a 60-mesh screen. The ground materials were then thoroughly mixed in a food mixer for 20 min, with 25 volume percent of water added to form a homogeneous mixture. This mixture was subsequently pelleted into 1.5 mm diameter pellets using a feed pelletizer (YSPM, manufactured by Yusheng Equipments Co., Ltd., Shanghai, China). The resulting pellets were air-dried at room temperature and then preserved at 4 °C for future utilization.

### 2.2. Fish, Cultured Conditions, and Sample Collection

*T. ovatus* juveniles (initial weight: about 30 g) were bought from a native hatchery and acclimated in floating cages (3.0 m × 3.0 m × 2.0 m) on the coast near Nan Ao Marine Biology Station of Shantou University for 2 weeks. The fish were fed a mixture of the three diets during the acclimation. Before the feeding trial, 270 fish of similar size were weighed individually, then casually distributed to 9 floating cages (1 m × 1 m × 1.5 m, 30 fish/cage), and each diet was randomly assigned to three cages. In the course of the 8-week culture experiment, fish were fed at 7:30 and 17:30 to obvious satiety. The culture conditions were as follows: temperature ranged from 27 °C to 30 °C, salinity fluctuated between 30‰ and 33‰, and dissolved oxygen maintained a level greater than 5 mg/L. The consumed diets of each cage were recorded each day, and dead fish were timely weighed and recorded.

After the feeding trial, a 24-h fasting period was implemented for the fish, followed by anesthesia using MS-222 (60 mg/L, Sigma, Livonia, MI, USA). Each net cage underwent individual counting and weighing of all fish. The feed intake was documented to assess the growth performance and feed utilization efficiency of the fish. Morphological indices were calculated based on measurements of body weight, length, viscera weight, and liver weight from three randomly selected fish per net. Muscle samples from six fish per cage were rapidly frozen in liquid nitrogen and stored at −80 °C for the analysis of proximate composition, fatty acid composition, and volatile compounds. Additionally, three fish from each cage were utilized to analyze edible quality and textural characteristics.

### 2.3. Proximate and Fatty Acid Compositions Analysis

According to the standard methods [18], the proximate composition of the experimental diets and muscle samples was determined. Briefly, the moisture was determined by drying the sample in an oven at 105 °C to constant weight. The determination of ash was obtained by incinerating samples in a muffle furnace at 550 °C for six hours. Crude protein was measured with a semi-automatic Kjeldahl analyzer (KDN-102C, Xianjian Instruments Co., Ltd., Shanghai, China), and Soxhlet extraction with petroleum ether was used to analyze crude lipid.

Total lipids were extracted from muscle tissues and dietary samples using a chloroform/methanol (*V*/*V*, 2:1) solution. This method ensures efficient extraction of a broad range of lipid classes by leveraging the polarity differences of the solvents. The mixture was homogenized thoroughly to facilitate lipid release from the biological matrices. Fatty acids present in the extracted lipids were then subjected to methylation. This process was carried out using boron trifluoride diethyl etherate (approximately 48%, Acros Organics, Waltham, MA, USA). The methylation reaction converts the fatty acids into their corresponding methyl esters, making them more volatile and suitable for gas chromatography analysis. The fatty acid methyl esters were analyzed using a gas chromatography system (GC, 7890B-5977A, Agilent Technologies Inc., Santa Clara, CA, USA). The following GC conditions were employed: the GC column was a Hewlett-Packard DB-WAX column (60 m × 250 μm × 0.25 μm), and the column chamber heating conditions were as follows: the initial temperature of the column chamber was 120 °C, and the maximum temperature was 250 °C. Inlet settings: The heater temperature was adjusted to 250 °C, and the carrier gas (high-purity nitrogen) flow rate was 1.4 mL/min. Detector conditions: The heater temperature was set to 250 °C, the airflow rate was 400 mL/min, the hydrogen gas flow rate was 40 mL/min, and the tail-blown nitrogen flow rate was 25 mL/min. The system was calibrated with commercial fatty acid standards obtained from Sigma to ensure accurate identification and quantification. Commercial fatty acid standards from Sigma were used to identify fatty acid methyl esters. These standards provide reference retention times and mass spectral data necessary for the accurate identification of the components in the sample. Calibration curves were generated to quantify the concentrations of the detected fatty acids with high precision.

### 2.4. Edible Quality and Textural Characteristics

The edible quality of muscle was estimated by cooking percentage (CP) and water holding capacity (WHC), whose calculation formulas are as follows: CP (%) = 100 × the weight of the muscle after cooking (g)/initial muscle weight before cooking (g); WHC (%) = 100 × [muscle weight before extrusion (g)-muscle weight after extrusion (g)]/muscle weight before extrusion (g). It was assessed by placing fresh muscle samples in heat-resistant plastic bags and reweighing after steaming at 100 °C for 5 min.

The adhesiveness, chewiness, cohesiveness, elasticity, gumminess, hardness, resilience, springiness, and tenderness of muscle were quantified by the Universal Texture Analyzer (Shanghai Tengba Instrument Technology Co., Ltd., Shanghai, China) based on our previous research [19]. The specific method is briefly described as follows: two muscles of identical dimensions (1.0 × 1.0 × 1.0 cm) were excised from the lateral aspect of the fish with a sharp scalpel for texture analysis. One side of the muscle samples was steamed in boiling water for five minutes to analyze the texture of the cooked muscle, while the other side was tested for the texture of the raw muscle. Texture analyzers are used to evaluate the texture of muscle samples in the TPA mode. This involved compressing the samples twice with an 8-mm cylindrical (2 mm/s), with a trigger force of 5 g, a retention time of 5 s, and a deformability of 60%.

### 2.5. Identification and Relative Quantification of Volatiles

Samples were removed from the −80 °C refrigerator and ground in liquid nitrogen. Each sample was mixed evenly, weighed to 0.2 g, and placed in a headspace bottle. 0.2 g of sodium chloride and 10 μL of internal standard solution were added to each bottle. The headspace bottle was then maintained at a constant temperature of 60 °C for 5 min. After that, the 120 µm DVB/CWR/PDMS extraction head was inserted into the bottle to carry out headspace extraction for 15 min. Next, the analysis was performed at 250 °C for 5 min, followed by GC-MS separation and identification. The volatile compounds were separated and detected using a GC-MS/MS (Agilent 8890-7000D).

The conditions for chromatography are as follows: DB-5MS capillary column (30 m × 0.25 mm × 0.25 μm, Agilent J&W Scientific, Folsom, CA, USA) is equipped using high purity helium (purity ≥ 99.99%) with a constant flow rate of 1.2 mL/min. The inlet temperature is set to 250 °C with a solvent delay of 3.5 min. Programmed temperature rise: 40 °C for 3.5 min, then increase to 100 °C at a rate of 10 °C per minute, followed by an increase to 180 °C at a rate of 7 °C per minute, then up to 280 °C at a rate of 25 °C per minute. Hold at 280 °C for 5 min. The specific conditions used in the mass spectrum are as follows: electron bombardment ion source (EI) at 230 °C, quaternary bar temperature at 150 °C, interface temperature at 280 °C, electron energy set to 70 eV, with the SIM scanning mode for ion detection. Qualitative and quantitative ion precision scanning was performed with the standard [20]

The odor activity value (OAV) of each volatile was evaluated by the ratio of the concentration of the volatile and its corresponding odor thresholds to investigate the contribution of each differential volatile compound to the overall odor profile [21]. The threshold values of volatiles used in the present study were obtained from the literature [14,22,23,24,25,26,27,28,29,30].

### 2.6. Statistic Analysis

The experimental data are expressed as mean ± standard error of the mean (SEM, *n* = 3). One-way analysis of variance (ANOVA) followed by Tukey’s multiple comparison test was utilized to compare the difference between dietary groups, with the significance set at *p* < 0.05. The analysis utilized SPSS v17.0 (SPSS Inc., Chicago, IL, USA). Hierarchical cluster analysis (HCA) of the volatile compounds among different dietary groups was conducted using ImageGP2 (Version 2) (http://www.bic.ac.cn/ImageGP, accessed on 20 February 2024). Partial square discriminant analysis (PLS-DA) and VIP scores were conducted using SIMCA 14.1 to identify discrepancies in muscle volatile compounds among dietary groups and to pinpoint key volatile compounds driving those differences. Additionally, correlation analysis between key differential volatile metabolites and fatty acids was also carried out using Origin 2020b.

## 3. Results

### 3.1. Growth and Morphometric Indices

The growth performance and morphological indexes of fish among the three dietary groups are shown in Table 1. No significant differences were observed in survival rate, feed conversion ratio, hepatosomatic index, viscerosomatic index, and condition factor among the D1-D3 groups (*p* > 0.05). However, compared to the FO group (D1), the final body weight, weight gain rate, and specific growth rate of the FO replacement groups (D2 and D3 groups) were lower; notably, those growth indicators of the D3 group exhibited a significant reduction (*p* < 0.05). While, the growth performance of fish in the D2 group was better than that in the D3 group, and there was no significant difference between the D2 group and the D1 group (*p* > 0.05).

### 3.2. Proximate Compositions of Muscle

**Figure 1:**
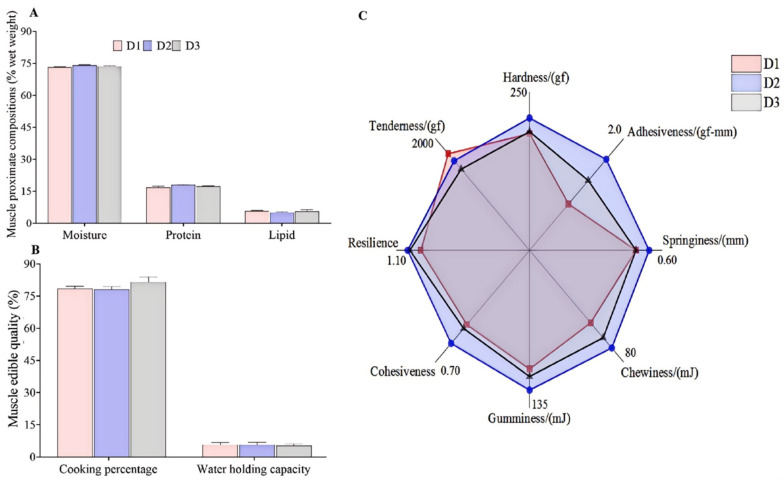
Proximate compositions (**A**), edible quality (**B**), and textural properties (**C**) of the muscle of *T. ovatus* fed different diets. Values are means ± SEM (*n* = 3, three fish per cage), and the detailed values are listed in Tables S1 and S2.

Figure 1A shows the proximate composition of fish muscle, which indicates no significant difference in the contents of moisture, protein, and lipid among the three dietary groups (*p* > 0.05). Although there were no statistical differences in the data of muscle proximate compositions, the D2 group had relatively higher protein contents and lower lipid contents than the D1 and D3 groups.

### 3.3. Edible Quality and Textural Characteristics of Muscle

There was no significant difference in muscle edible quality (CP and WHC) among the three groups (Figure 1B, *p* > 0.05), while the muscle adhesiveness and cohesiveness in the diet D2 group were significantly enhanced compared with the diets D1 or D3 groups (Figure 1C, *p* < 0.05). There were no significant differences in the other texture parameters among the three groups, though. The value of muscle springiness, chewiness, and resilience in the D2 group was relatively greater than in the D1 and D3 groups.

### 3.4. Differentiation of T. ovatus Fed Different Diets Based on the Fatty Acid Profiles

**Figure 2:**
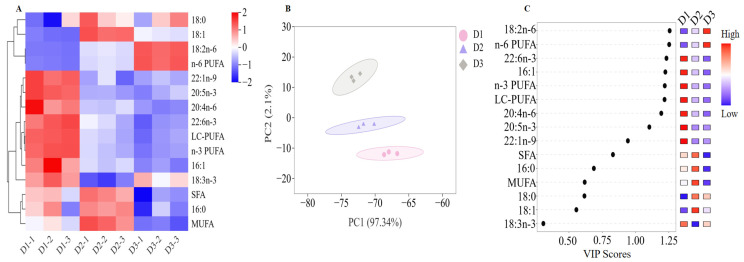
Muscle fatty acid compositions of the *T. ovatus* fed with different diets. (**A**) clustering heatmap of main fatty acids; (**B**) partial square discriminant analysis (PLS-DA) of identified fatty acid compositions; (**C**) VIP scores of identified main fatty acid compositions. The detailed values are listed in Table S3.

As shown in Table S2, the primary fatty acids of *T. ovatus* muscle are palmitic acid (PA, 16:0), oleic acid (OA, 18:1n-9), linoleic acid (LA, 18:2n-6), and DHA (22:6n-3), which is a classic fatty acid profile in marine fish tissue. The fatty acid profiles of muscle (especially for LC-PUFA and its substrates) of most marine fish generally reflect the fatty acid composition of the corresponding diet. The contents of arachidonic acid (ARA, 20:4n-6), EPA, DHA, LC-PUFA of the muscle in the D2 and D3 groups are significantly lower than those in the D1 group (FO diets, Figure 2A) (*p* < 0.05), whereas content of ALA and LA has the opposite trend. Notably, compared to diet D3, diet D2 contained lower LC-PUFA levels and high SFA contents, while the contents of LC-PUFA in the muscle of the diet D2 and D3 groups were comparable (Figure 2A).

Based on the analysis of PLS-DA of fatty acid compositions among the three dietary groups, three well-separated clusters were detected in the PCA score plot (Figure 2B). Furthermore, the analysis with the identical screening criteria (VIP > 1.0, *p* < 0.05) confirmed eight significant contributors to the differential fatty acid composition in fish fed with diets D1, D2, or D3, including LA, n-6 PUFA, DHA, 16:1, n-3 PUFA, LC-PUFA, ARA, and EPA (Figure 2C). In the present study, a high content of DHA, 16:1, n-3 PUFA, LC-PUFA, ARA, and EPA was detected in the D1 group, while a high content of LA, n-6 PUFA was detected in the D3 group (*p* < 0.05).

### 3.5. Overall Volatiles of T. ovatus Fed Different Diets

**Figure 3:**
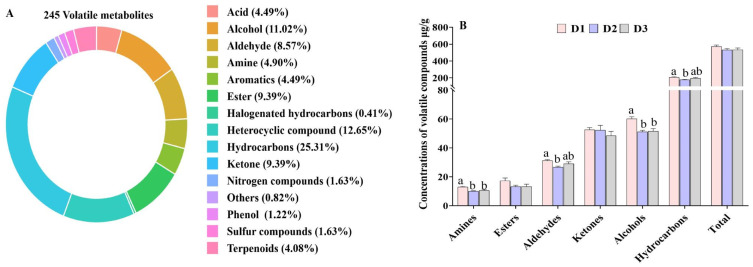
The overall composition (**A**) and relative contents of volatile metabolites (**B**) in the muscle of *T. ovatus* fed with different diets. The detailed values are listed in Tables S4 and S5. The bars (in (**B**) with different superscripts mean significant difference (*p* < 0.05).

A total of 245 volatile compounds, including 62 hydrocarbons, 31 heterocyclic compounds, 28 alcohols, 24 aldehydes, 23 ketones, 23 esters, 12 acids, 12 amines, 10 aromatics, 4 nitrogen compounds, and 20 other compounds, were identified in the muscle samples (Figure 3A). The predominant odor-active compounds in *T. ovatus* muscle were alcohols and ketones, followed closely by aldehydes.

Additionally, the content of alcohols, aldehydes, and amines was significantly lower in the D2 and D3 groups in contrast to those in the D1 group (*p* < 0.05), while the content of total esters and ketones did not demonstrate a significant difference among the three groups (Figure 3B). Among the top ten highest contents of aldehydes, the high contents of butanal, citral, furaldehyde, 2,4-octanedienal, (z)-3-hexena, and benzeneacet aldehyde were detected in the D1 group, which is similar with that of total aldehydes, while the content of 3-methyl butyraldehyde, perillyl aldehyde, and decanal were comparable among the three groups (Figure S1A). Notably, high 3,4,5-trimethyl-2-cyclopenten-1-one, 2-methyl-cyclopentanone, and 3-octene-2 ketone contents were detected in the D1 group in comparison with those in the D2 and D3 groups, which is different from the profile changes of total ketones (Figure S1B). In terms of alcohol profiles, 3-methyl butanol accounted for nearly 90% of the total alcohols present, with a higher concentration observed in the D1 group compared to the D2 and D3 groups (Figure S1C). The high content of aldehydes, alcohols, and some ketones found in the D1 group indicates that the muscle flavor of farmed *T. ovatus* fed FO diets is more intense.

### 3.6. Differentiation of T. ovatus Fed Different Diets Based on the Volatiles

**Figure 4:**
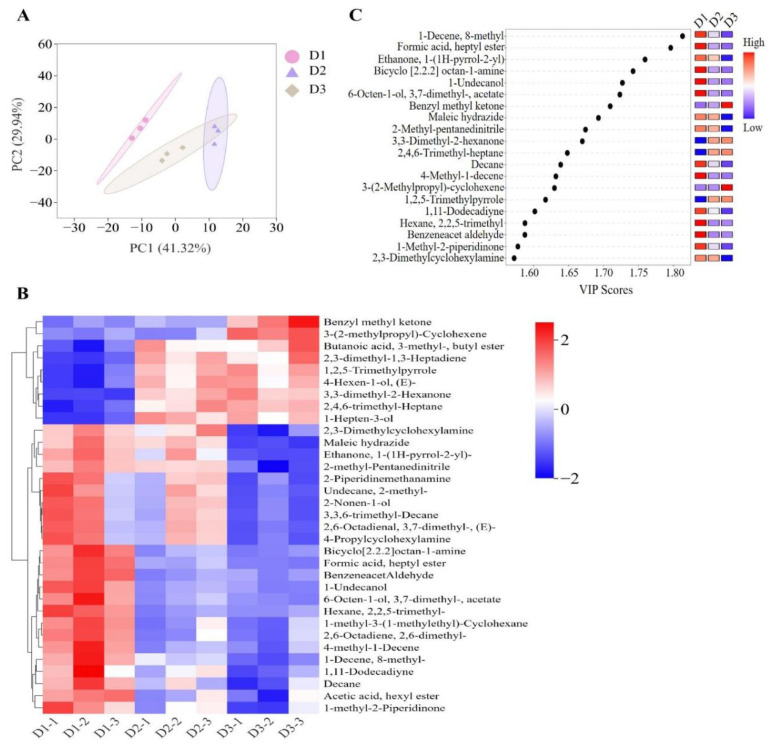
Muscle identified volatile flavors of the *T. ovatus* fed with different diets. (**A**) partial square discriminant analysis (PLS-DA) of identified volatile flavors; (**B**) hierarchical cluster analysis of top thirty differential volatile flavors; (**C**) VIP scores of dentified key volatile flavors. The detailed values are listed in Table S6.

The analysis of PLS-DA was applied to provide an overall picture of volatile compounds in muscle among the three groups (Figure 4A) based on the concentrations of 245 volatile compounds. The PLS-DA analysis showed that the D1 group was clearly separated from the other two groups, with a cumulative contribution rate of 71.26% (PC1 and PC2 contributing 41.32% and 29.94%, respectively). The profiles of volatile compounds were clearly clustered into three groups, indicating that the muscle volatile compounds of *T. ovatus* fed different diets could be distinctly separated. Additionally, 108 differential volatile compounds (VIP > 1) are identified among the different groups (Table S6). The HCA was conducted based on the top thirty differential volatile flavors to further elucidate the variations in volatile compound profiles among the three dietary groups, resulting in their classification into two main clusters (Figure 4B). Cluster I was further divided into two subgroups. One subgroup showed high content of benzyl methyl ketone and 3-(2-methylpropyl)-cyclohexene in the D3 group, while another subgroup of *T. ovatus* fed diets D2 and D3 containing plant oil gathered some high volatiles such as butanoic acid, 3-methyl-, butyl ester, 2,3-dimethyl-1,3-heptadiene, 1,2,5-trimethylpyrrole, 4-hexen-1-ol (E), 3,3-dimethyl-2-hexanone, 2,4,6-trimethyl-heptane, and 1-hepten-3-ol (Figure 4B). Similarly, cluster II was also divided into two subgroups. One subgroup showed high 2,3-dimethylcyclohexylamine, maleic hydrazide, etc., in the D1 and D2 groups, and high bicyclo [2.2.2] octan-1-amine, formic acid heptyl ester, etc., were detected in D1 group (Figure 4B).

Among the differential volatile compounds, 20 important contributors were further identified for volatile differentiation of fish-fed different diets using identical screening criteria (VIP > 1.0, *p* < 0.05) (Figure 4C). For example, significantly high contents of 1-decene-8-methyl, formic acid heptyl ester, bicyclo [2.2.2] octan-1-amine, 1-undecanol, and 6-octen-1-ol 3,7-dimethyl-acetate, 4-methyl-1-decene, hexane 2,2,5-trimethyl, and benzeneacet aldehyde were detected in the FO diet groups (enriched in LC-PUFA) compared with those in the D2 and D3 diet groups (containing low LC-PUFA), whereas the opposite was true for 3,3-dimethyl-2-hexanone, 2,4,6-trimethyl-heptane, and 1,2,5-trimethylpyrrole (Figure 4C). Furthermore, fish-fed diets with high n-6 PUFA (diet D3) exhibited elevated concentrations of benzyl methyl ketone and 3-(2-methylpropyl)-cyclohexene, while their levels of ethanone, 1-(1H-pyrrol-2-yl), maleic hydrazide, 2-methyl-pentanedinitrile, and 2,3-dimethylcyclohexylamine showed the opposite trend (Figure 4C).

**Figure 5:**
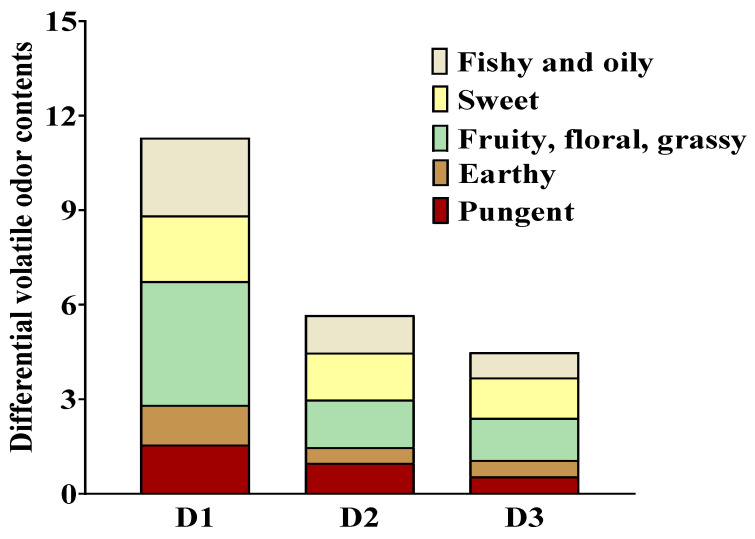
Sensory property of the muscle of *T. ovatus* fed with different diets. The volatile odors were collected from the differential volatile compounds related to fatty acids. Detailed values and odor descriptions were listed in Table S6 and Table S7, respectively.

**Figure 6:**
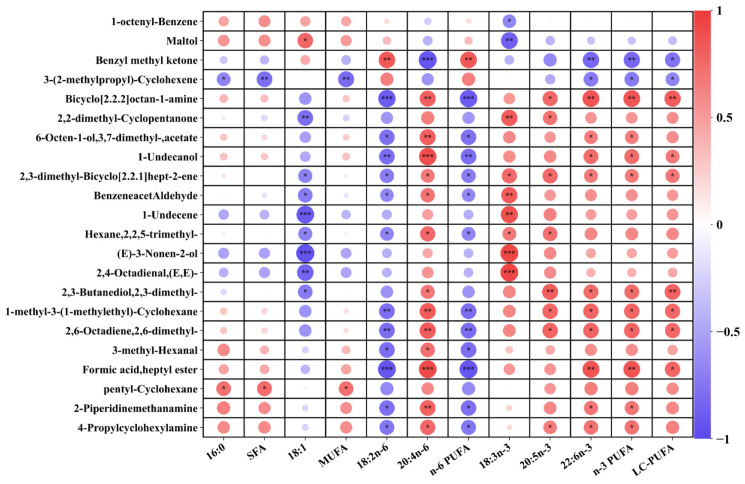
Correlation analysis between key differential volatile metabolites and fatty acids. The “*” indicates significant correlations (*p* < 0.05), while “**” (*p* < 0.01) and “***” (*p* < 0.001) indicate extremely significant correlations.

The contribution of volatile compounds to the odor profile of samples does not depend on their concentrations but on their corresponding OAV [21]. Volatiles with an OAV greater than 1 generally play a significant role in the odor of samples, while those with an OAV less than 1 have a lesser impact [29,30]. The analysis presented in Table S7 revealed the presence of 24 key differential volatile compounds (>1.0 μg/g) among the four dietary groups. However, only two volatiles, namely 3,4,5-trimethyl-2-cyclopenten-1-one (OAV = 4.18~5.02, sweet, fruity) and 2(3H)-furanone-5-ethenyldihydro-5-methyl (OAV = 0.69~1.10, caramel, maple), exhibited OAV greater than 1 and significantly contributed to the distinct aroma profiles observed in *T. ovatus* fed diets with different lipid sources. Of all the differential volatiles identified, the OAV and type of volatiles with pleasant flavor (sweet, fruity, caramel, maple, mushroom, and grassy) were higher than those with off-flavor (pungent, amine, burnt, oily, and fishy). Additionally, a high abundance of pleasant flavors was detected in the fish-fed diet D1 (Figure 5, Table S7). Notably, the contents of total volatile compounds and pleasant flavor compounds in the D2 group were greater compared with the D3 group (Figure 6, Table S7). The results indicated that FO substitution impairs the odor characteristics of farmed *T. ovatus*, particularly its pleasant flavor. Thus, when substituting FO with palm and rapeseed oils, the impact on the odor profile is comparatively reduced compared to substituting FO with soybean oil.

### 3.7. Correlation Analysis Between Key Differential Volatiles and Fatty Acid

The correlation between key differential volatile compounds (concentration > 1.0 μg/g) and fatty acid is presented in Figure 6. The muscle n-3 PUFA and LC-PUFA contents exhibit a positive correlation with the OAV of almost all key differential volatile compounds, except for 2-methoxythiophene. This correlation is particularly evident in the case of 2,3-butanediol 2,3-dimethyl, (E)-3-Nonen-2-ol, pentadecane, 2-methyl-cyclopentanone, and (1-methylethylidene)-cyclohexane. The muscle LA and n-6 PUFA contents exhibit a positive correlation only with the OAV of 2-methoxythiophen, while showing a negative correlation with the others. However, there is a limited correlation between the muscle SFA contents and the OAV of volatile compounds, except for acetamide and 2-methyl-3-pentanone. The results confirmed that the overall volatile compounds from lipid oxidation are determined by the degree of unsaturation of fatty acids.

## 4. Discussion

Like other marine fish, *T. ovatus* has limited ability to synthesize LC-PUFA, such as DHA and EPA, which are essential fatty acids (EFA). Therefore, their feed needs to be supplemented with FO rich in HUFA to meet their healthy growth requirements [31]. In order to reduce the dependence of marine fish compound feed on limited and expensive resources, aquatic scientists have carried out a large number of studies on the substitution of fish oil with terrestrial animal and plant oils [2,4]. The results show that appropriate substitution of FO has no negative impact on growth, but excessive substitution significantly reduces fish growth [5,6]. Similarly, previous studies have found that excessive substitution of terrestrial plant and animal oils for dietary fish oil can easily result in insufficient EFA intake, which affects the normal growth of *T. ovatus* [12,13,14,31]. The above results confirm that *T. ovatus* has a limited ability to synthesize n-3 LC-PUFAs required for growth from ALA [31]; thus, FO (rich in n-3 LC-PUFAs) must be added to its feed in sufficient quantities or 1.24–1.73% n-3 LC-PUFAs [32]. Notably, in this study, the growth performance of fish fed with D2 feed was superior to that of fish fed with D3 feed, while no significant difference was observed between the D2 group and the FO group. The reason for the above results may be that the SFA levels in diet D2 are higher than in diet D3, and a sufficient supply of SFA in the diets can reduce the catabolism of n-3 LC-PUFA, which is conducive to the n-3 LC-PUFA sparing [33]. Additionally, the dietary ratio of n-3/n-6 PUFA in diet D2 surpasses that in diet D3, thereby facilitating the normal growth of fish [34,35]. Consequently, compared to soybean oil, the combination of palm and rapeseed oils proves advantageous in enhancing the application efficacy of a low FO diet in *T. ovatus*.

Although appropriate FO substitution has no negative impact on the growth performance of marine fish when the EFA requirements are met, it has a relatively substantial impact on muscle quality [5,6]. This study revealed that the muscle lipid content in n the D2 group decreased by 6.77% compared to that in the D1 group, which is consistent with our previous findings in *T. ovatus* [16]. Similarly, the FO substitution-related studies indicated that diets with relatively high MUFA and low n-6 PUFA are beneficial in promoting protein retention and decreasing lipid deposition in the fish muscle [16,36,37]. The above results suggested that the muscle protein and lipid contents of cultured fish fed low FO diets can be reasonably regulated by dietary lipid and fatty acid nutrition.

Muscle lipid content exhibits an inverse relationship with muscle texture quality. Specifically, a reduction in muscular lipid content correlates with improved textural attributes such as hardness, chewiness, and cohesiveness, ultimately enhancing the overall taste profile [7]. The studies of grass carp (*Ctenopharyngodon idella*), Atlantic salmon (*Salmo salar*), and brook charr (*Salvelinus fontinalis*) found that a modification of muscle texture in FO substitution treatments was observed compared with the fish oil treatment [7]. While, FO substitution had little or no effect on the muscle texture profile of certain fish species, such as Atlantic cod (*Gadus morhua*) and tiger puffer (*Takifugu rubripes*) [7], which is similar to the comparable muscle texture profile of *T. ovatus* fed diet D1 and diet D3. Notably, the fish fed diets D2 demonstrated superior performance in terms of texture quality compared with that of fish fed with diets D3. In comparison with the D3 diet, the D2 diet contained the high SFA level. Consequently, it is hypothesized that the enhanced textural properties observed in the D2 group may be attributed to their higher intake of SFA. In an investigation of Japanese sea bass (*Lateolabrax japonicus*), it was found that fish with higher concentrations of SFA in their muscles exhibited greater texture profile compared to those with higher levels of MUFA and PUFA [38]. The results suggest that the observed difference may be due to varying fatty acid requirements and utilization among species, which means the muscle texture of cultured fish can be improved by reasonable fatty acid nutrition.

The composition and content of fatty acids in fish muscle are important indicators for evaluating the nutritional quality of fish muscle. In this study, the content of LC-PUFA in the muscle of *T. ovatus* in the control group was significantly higher than that in the two FO substitution groups. Among the FO substitution groups, the D3 group (rich in n-6 PUFA) had higher levels of LA and n-6 PUFA in the muscle, which is not conducive to consumers maintaining the balance of n-6/n-3 PUFA [39,40]. Compared to the D3 group, the D2 group have comparable muscle LC-PUFA contents, while, the ratio of n-6 to n-3 PUFA in the latter is more balanced, and the nutritional quality of fatty acids is more balanced as well [39,40]. Notably, compared to diet D3, diet D2 contained lower LC-PUFA levels and high SFA contents, which findings confirmed that SFA exhibits an LC-PUFA-sparing effect [31], which is beneficial for improving the fatty acid nutrition of fish when fed with FO substitution diets.

In addition to nutritional quality, flavor constitutes a critical criterion for evaluating meat quality. The primary volatile compounds in fish are categorized into aldehydes, ketones, alcohols, and esters [10]. In this study, a total of 245 volatile compounds, including 28 alcohols, 24 aldehydes, 23 ketones, 23 esters were detected in the *T. ovatus*, which is more abundant than the volatile compounds identified from rainbow trout (*Oncorhynchus mykiss*), yellowfin tuna (*Thunnus albacores*), large yellow croaker (*Larimichthys crocea*), and common carp [33,41,42,43]. Most of these potential volatiles were alcohols, aldehydes, and ketones, which were generated during the autoxidation of PUFA [44]. The high content of aldehydes, alcohols, and some ketones found in the D1 group indicates that the muscle flavor of farmed *T. ovatus* fed FO diets is more intense.

Dietary lipid sources not only affect the nutritional quality of fish muscle, but also influence their flavor characteristics. Existing research has established that lipids serve as crucial precursors in the metabolic synthesis of odor compounds [10]. The muscle volatile compounds of swimming crab (*Portunus trituberculatus*) and turbot (*Scophthalmus maximus*) can be differentiated between the dietary fish oil groups and plant oil/beef tallow groups [17,45]. Similarly, the profiles of volatile compounds of *T. ovatus* fed different diets were distinctly separated into three groups, and 20 differential volatile compounds were further identified among the three groups. Inconsistently, relatively low levels of differential volatile compounds were detected in gilthead sea bream (*Sparus aurata*), swimming crab, and tilapia fed with diets composed of dietary lipids from different sources [46,47]. This may be due to the different detection technology for volatile compounds or the higher sensitivity of *T. ovatus* to dietary lipid sources compared to other aquatic species.

Accumulation of studies indicated that most volatile compounds, such as aldehydes, ketones, alcohols, and hydrocarbons, are derived from lipid and fatty acids [10,48,49,50,51,52]. The saturated aldehydes (hexanal and nonanal) principally arise from the oxidation of n-6 PUFA (LA and ARA), propionaldehydes are derived from ALA [10,49], alcohols, such as 1-octen-3-ol, are formed by the oxidation of LA and ARA, 1-hexanol originates from MUFA (oleic acid and palmitic acid) [10]. However, in livestock products, the concentrations of n-3 PUFA oxidation products are positively correlated with the saturated and unsaturated aldehydes, alcohols, and ketones [51,52]. Similarly, high positive correlations were observed between n-6 PUFA-derived aldehydes and the n-6 PUFA contents in tench (*Tinca tinca* L.) fed high n-6 PUFA diets [48]. Compared to the FO group (D1), the concentrations of total volatile compounds and pleasant flavor compounds were relatively lower in the FO-replacement feed groups (D2 and D3). However, the aforementioned indicators were higher in group D2 compared to group D3. Additionally, the correlation between the differential volatile compounds and fatty acids identified in this study follows this order: n-3 PUFA and LC-PUFA, followed by SFA, and then n-6 PUFA. The results indicated that FO substitution impairs the odor characteristics of farmed *T. ovatus*, particularly its pleasant flavor [48]. Thus, when substituting FO with palm and rapeseed oils, the impact on the odor profile is comparatively reduced compared to substituting FO with soybean oil.

## 5. Conclusions

This study investigated the effects of dietary lipid sources on growth and muscle quality, including proximate composition, texture, edible quality, fatty acid profiles, and volatile compounds in *T. ovatus*. FO diets resulted in better growth, high contents of muscle LC-PUFA, lipid, and pleasant flavors of *T. ovatus*. At the same time, FO substitution diets with palm and rapeseed oils are more beneficial to muscle texture than FO diets. Among the FO substitution groups, palm and rapeseed oils presented better growth performances, muscle protein contents, texture, fatty acid nutrition, and volatile compounds, while FO substitution diets with soybean oil exhibited diminished growth and muscle quality. These findings offer new insights into enhancing the muscle quality of farmed *T. ovatus* through dietary fatty acid manipulation within the context of modern aquaculture utilizing low FO diets.

## Figures and Tables

**Table 1:** Growth performance and morphometric indices of *T. ovatus* fed different diets.

	Dietary Groups		
	D1	D2	D3
Initial body weight/g	39.33 ± 0.15	39.56 ± 0.06	39.88 ± 0.06
Final body weight/g	151.69 ± 1.98 ^a^	142.63 ± 3.99 ^ab^	133.67 ± 3.64 ^b^
Growth gain rate/%	385.53 ± 24.83 ^a^	360.51 ± 40.52 ^ab^	326.77 ± 31.85 ^b^
Specific growth rate/% d^−1^	2.33 ± 0.03 ^a^	2.21 ± 0.06 ^ab^	2.08 ± 0.05 ^b^
Survival rate/%	97.78 ± 1.11	98.89 ± 1.11	96.67 ± 1.93
Feed coefficient	1.54 ± 0.04	1.64 ± 0.09	1.65 ± 0.09
Hepatosmatic index/%	1.31 ± 0.16	1.34 ± 0.09	1.55 ± 0.09
Viscerosmatic index/%	7.2 ± 0.29	6.5 ± 0.15	6.93 ± 0.24
Condition factor/g cm^−3^	3.07 ± 0.11	3.29 ± 0.06	3.28 ± 0.09

Values are means ± SEM (*n* = 3, three fish per cage), and different letters in the same rows represented significant differences among the different groups (*p* < 0.05).

## Data Availability

The original contributions presented in this study are included in the article/Supplementary Material. Further inquiries can be directed to the corresponding author.

## Supplementary Materials

The following supporting information can be downloaded at https://www.mdpi.com/article/10.3390/foods14050788/s1, Figure S1: Relative contents of volatile metabolites in the muscle of *T. ovatus* fed with different diets. Table S1: Composition and nutrient levels of experimental diets (% dry weight); Table S2: Proximate compositions, edible quality, and textural properties of the muscle of *T. ovatus* fed different diets; Table S3: Muscle fatty acid compositions of the *T. ovatus* fed different diets (%total fatty acids); Table S4: Volatile compounds identified in muscle of the *T. ovatus* fed different diets; Table S5: Relative concentrations of volatile compounds of the *T.* ovatus muscle fed different diets (μg/g); Table S6: Contents of differential volatile compounds (VIP > 1) in the muscle of *T. ovatus* fed different diets (μg/g); Table S7: Concentrations and odor activity values of differential volatiles in the muscle of *T. ovatus* fed different diets.

## Abbreviations

ALA: α-linolenic acid; ANOVA, Analysis of variance; ARA, arachidonic acid; CP, cooking percentage; DHA, docosahexaenoic acid; DVB/CWR/PDMS, divinylbenzene/carbon wide range/polydimethylsiloxane; EFA, essential fatty acids; EPA, eicosapentaenoic acid; EI, electron bombardment ion; Fads, fatty acyl desaturase; FO, fish oil; FAME, fatty acid methyl esters; GC-MS, gas chromatography-mass spectrometer; HCA, hierarchical cluster analysis; LA, linoleic acid; LC-PUFA, long-chain polyunsaturated fatty acid; MUFA, monounsaturated fatty acid; PLS-DA, partial square discriminant analysis; PUFA, polyunsaturated fatty acid; SFA, saturated fatty acid; UFA, unsaturated fatty acids; WHC, water holding capacity.
