# Supplementary material for: Rapeseed and Palm Oils Can Improve the Growth, Muscle Texture, Fatty Acids and Volatiles of Marine Teleost Golden Pompano Fed Low Fish Oil Diets
Source: Foods. 2025 Feb 25;14(5):788. doi: 10.3390/foods14050788 (PMC11899666; doi:10.3390/foods14050788)
Supplement: Supplementary file 1 [file foods-14-00788-s001.zip › foods-3319164-supplementary.pdf]

Supplementary materials

Figure S1. Relative contents of volatile metabolites in muscle of *T. ovatus* fed with different diets.

Table S1. Composition and nutrient levels of experimental diets (% dry weight).

Table S2. Proximate compositions, edible quality and textural properties of the muscle of *T. ovatus* fed different diets.

Table S3. Muscle fatty acid compositions of the *T. ovatus* fed different diets (% total fatty acids).

Table S4. Volatile compounds identified in muscle of the *T. ovatus* fed different diets.

Table S5. Relative concentrations of volatile compounds of the *T. ovatus* muscle fed different diets (μg/g).

Table S6. Contents of differential volatile compounds (VIP > 1) in the muscle of *T. ovatus* fed different diets (μg/g).

Table S7. Concentrations and odor activity values of differential volatiles in the muscle of *T. ovatus* fed different diets.

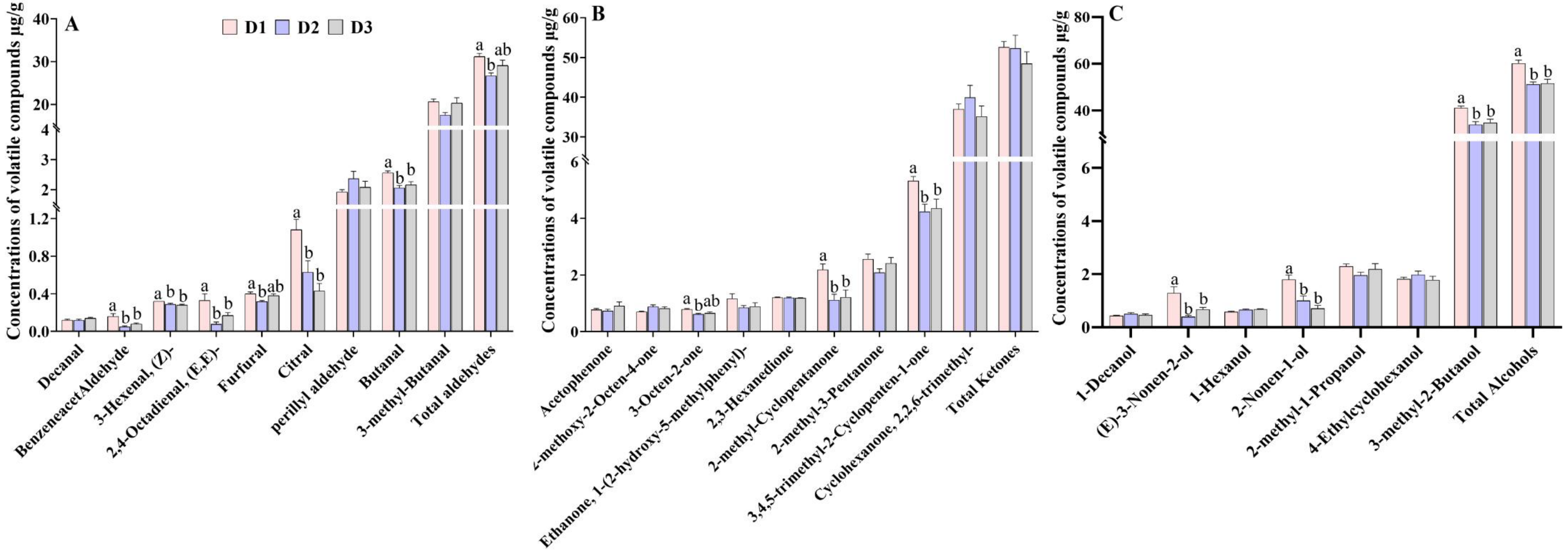

Figure S1. Relative contents of volatile metabolites in muscle of *T. ovatus* fed with different diets.

Table S1. Composition and nutrient levels of experimental diets (% dry weight).

Items	Dietary treatments		
	D1	D2	D3
Ingredients			
Fishmeal	18.00	18.00	18.00
Blend protein[1]	46.00	46.00	46.00
Fish oil	8.00	3.2	3.2
Rapeseed oil	/	2.4	/
Palm oil	/	2.4	/
Soybean oil	/	/	4.8
Bread flour	17.00	17.00	17.00
Sprayed corn skin	6.20	6.20	6.20
Others[2]	4.80	4.80	4.80
Proximate composition (%)			
Dry matter	92.70	92.44	92.56
Crude protein	49.56	49.18	49.29
Crude lipid	12.05	12.21	12.21
Ash	9.09	9.24	9.44
Fatty acids composition (mg/g)			
14:0	5.17	3.89	2.76
16:0	22.63	20.68	17.33
18:0	6.17	5.53	3.73
16:1n-9	6.09	3.48	3.23
18:1n-9	17.25	25.61	20.91
22:1n-9	0.50	3.27	0.51
18:2n-6	12.63	17.98	30.01
18:3n-3	4.10	6.24	3.51
20:4n-6	2.12	0.79	0.70
20:5n-3	7.48	3.58	3.23
22:6n-3	10.67	6.96	7.08
SFA	30.66	28.78	24.30
MUFA	25.62	27.58	26.65
n-3 PUFA	24.25	17.46	14.87
n-6 PUFA	15.75	20.87	32.70
LC-PUFA	20.35	11.14	11.72

[1] Blend protein was composed of chicken meal:soy protein concentrate:corn gluten meal = 5:5:7.

[2] Others: including vitamin and premix (2 %), L-lysine (0.5 %), DL-methionine (0.5 %), choline chloride (0.3 %), calcium dihydrogen phosphate (1.5 %). All the dietary ingredients were provided by Yangjiang Haiyi Biotechnology Co., LTD.

Table S2. Proximate compositions, edible quality and textural properties of the muscle of *T. ovatus* fed different diets.

Items	Dietary treatments		
	D1	D2	D3
	Proximate composition (% wet weight)		
Moisture	73.24 ± 0.11	74.11 ± 0.34	73.62 ± 0.22
Protein	16.76 ± 0.69	18.00 ± 0.22	17.27 ± 0.32
Lipid	5.66 ± 0.41	5.05 ± 0.28	5.52 ± 0.88
	Edible quality		
CP /%	78.4 ± 1.17	78.1±1.27	81.45±2.45
WHC /%	5.67 ± 1.15	5.67±1.23	5.33±0.76
	Textural properties		
Hardness /(gf)	197.88 ± 5.07	217.63 ± 4.66	200.38 ± 6.42
Adhesiveness /(gf-mm)	0.83 ± 0.21^{b}	1.63 ± 0.21^{a}	1.25 ± 0.10^{b}
Springiness /(mm)	0.58 ± 0.01	0.61 ± 0.01	0.58 ± 0.01
Chewiness /(mJ)	68.99 ± 2.54	84.95 ± 1.96	71.31 ± 3.51
Gumminess /(mJ)	118.89 ± 3.47	137.55 ± 2.67	121.95 ± 4.97
Cohesiveness	0.60 ± 0.01^{b}	0.68 ± 0.01^{a}	0.61 ± 0.01^{b}
Resilience	0.99 ± 0.05	1.05 ± 0.04	1.04 ± 0.02
Tenderness /(gf)	1743.82 ± 29.30^{a}	1623.41 ± 25.07^{ab}	1481.98 ± 55.01^{b}

Results are presented as the mean ± SEM (n = 3). Within each row, means without sharing a common letter are significantly different ($P < 0.05$), those lacking letters indicate no significant difference ($p > 0.05$). CP, cooking percentage; WHC, water holding capacity.

Table S3. Muscle fatty acid compositions of the *T. ovatus* fed different diets (% total fatty acids).

Main fatty acid compositions	Dietary treatments		
	D1	D2	D3
16:0	22.56±0.55[ab]	23.39±0.16[a]	20.44±1.07[b]
18:0	5.55±0.23	6.55±0.3	5.75±0.23
16:1n-9	5.63±0.45[a]	3.75±0.04[b]	2.87±0.2[b]
18:1n-9	22.84±0.17[b]	30.73±0.28[a]	24.95±1.13[b]
22:1n-9	3.65±0.13[a]	1.21±0.2[b]	1.28±0.09[b]
18:2n-6	8.09±0.1[c]	13.64±0.34[b]	21.88±1.21[a]
18:3n-3	3.62±0.07[a]	2.32±0.22[b]	3.19±0.15[a]
20:4n-6	0.79±0.07[a]	0.43±0.01[b]	0.32±0.02[b]
20:5n-3	3.20±0.09[a]	1.15±0.03[b]	1.07±0.16[b]
22:6n-3	10.11±0.27[a]	6.69±0.24[b]	5.02±0.31[c]
SFA	31.84±0.35[a]	33.28±0.36[a]	29.05±1.04[b]
MUFA	33.08±0.37[a]	35.69±0.34[a]	29.1±1.38[b]
n-3 PUFA	19.02±0.28[a]	10.58±0.3[b]	8.59±0.54[c]
n-6 PUFA	9.61±0.19[c]	15.21±0.39[b]	24.11±1.32[a]
LC-PUFA	14.32±0.12[ab]	8.33±0.42[b]	8.41±1.73[a]

Results are presented as the mean ± SEM (n = 3). Within each row, means without sharing a common letter are significantly different ($p < 0.05$).

Table S4. Volatile compounds identified in muscle of the *T. ovatus* fed different diets.

Volatile compounds (245)	Formula	RI[1]	CAS[2]
Aldehyde (24)			
Nonanal	C9H18O	1104	124-19-6
4-(1-methylethyl)-Benzaldehyde	C10H12O	1230	122-03-2
2,6-Nonadienal, (E, Z)-	C9H14O	1120	557-48-2
Decanal	C10H20O	1204	112-31-2
2,6,6-trimethyl-1-Cyclohexene-1-carboxaldehyde	C10H16O	1204	432-25-7
1-Cyclohexene-1-carboxAldehyde, 4-(1-methylethenyl)-	C10H14O	1207	2111-75-3
(E)-2-Octenal	C8H14O	1013	2548-87-0
Heptanal	C7H14O	905	111-71-7
(E)-4-Heptenal	C7H12O	913	929-22-6
3-methyl-Butanal	C5H10O	643	590-86-3
Furfural	C5H4O2	831	98-01-1
3-Hexenal, (Z)-	C6H10O	814	6789-80-6
2-Hexenal	C6H10O	814	505-57-7
2-Undecenal	C11H20O	1311	2463-77-6
2-Undecenal, E-	C11H20O	1311	53448-07-0
3-methyl-Hexanal	C7H14O	841	19269-28-4
ParAldehyde	C6H12O3	870	123-63-7
2,4-Octadienal, (E, E)-	C8H12O	1021	30361-28-5
BenzeneacetAldehyde	C8H8O	1081	122-78-1
Butanal	C4H8O	607	123-72-8
Citral	C10H16O	1174	5392-40-5
2,6-Octadienal, 3,7-dimethyl-, (E)-	C10H16O	1174	141-27-5
2-hydroxy-BenzAldehyde	C7H6O2	1203	90-02-8
2-Decenal, (Z)-	C10H18O	1252	2497-25-8
Ketones (23)			
3-Hexanone-2,2,4,4-d4	C6H8D4O	780	24588-54-3
2-methyl-3-Hexanone	C7H14O	789	7379-12-6
3-Octen-2-one	C8H14O	960	1669-44-9
6-Propenylbicyclo [3.1.0]hexan-2-one	C9H12O	1061	75283-46-4
Cyclohexanone, 2,2,6-trimethyl-	C9H16O	1086	2408-37-9
Benzyl methyl ketone	C9H10O	1128	103-79-7
3,3-dimethyl-2-Hexanone	C8H16O	868	26118-38-7
2,3-Hexanedione	C6H10O2	786	3848-24-6
3,5-Octadien-2-one	C8H12O	968	38284-27-4
3-Octanone	C8H16O	986	106-68-3
Acetyl valeryl	C7H12O2	989	96-04-8
3,4,5-trimethyl-2-Cyclopenten-1-one	C8H12O	965	55683-21-1
2-Undecanone	C11H22O	1251	112-12-9

Ethanone, 1-(2-hydroxy-5-methylphenyl)-	C9H10O2	1363	1450-72-2
2,5-Dimethylcyclohexanone	C8H14O	1013	932-51-4
Acetophenone	C8H8O	1029	98-86-2
2-methyl-Cyclopentanone	C6H10O	832	1120-72-5
3-methyl-Cyclopentanone	C6H10O	832	1757-42-2
2,5,6-trimethyl-4-Hepten-3-one	C10H18O	1008	16466-21-0
6-methyl-2,4-Heptanedione	C8H14O2	1024	3002-23-1
2,2-dimethyl-Cyclopentanone	C7H12O	905	4541-32-6
2-methyl-3-Pentanone	C6H12O	690	565-69-5
2-methoxy-2-Octen-4-one	C9H16O2	1113	24985-48-6
Alcohols (28)			
(1R,2S,4r)-4-((E)-prop-1-en-1-yl) cyclopentane-1,2-diol	C8H14O2	1244	1010481-89-9
2,3,4-Trimethyl-1-pentanol	C8H18O	867	6570-88-3
1-Dodecanethiol	C12H26S	1518	112-55-0
1-Decanol	C10H22O	1258	112-30-1
2-methoxy-Ethanol	C3H8O2	638	109-86-4
2-Nonen-1-ol	C9H18O	1167	22104-79-6
2-phenoxy-1-Propanol	C9H12O2	1247	4169-04-4
1-Hexanol	C6H14O	860	111-27-3
3-methyl-2-Butanol	C5H12O	616	598-75-4
1-Octen-3-ol	C8H16O	969	3391-86-4
2-Octen-1-ol, (E)-	C8H16O	1067	18409-17-1
1,6-Heptadien-4-ol	C7H12O	860	2883-45-6
4-methyl-3-Penten-1-ol	C6H12O	845	763-89-3
2,2,4-trimethyl-1,3-Pentanediol	C8H18O2	1073	144-19-4
2,3-Butanediol, 2,3-dimethyl-	C6H14O2	801	76-09-5
4-Hexen-1-ol, (E)-	C6H12O	868	928-92-7
1-Hepten-3-ol	C7H14O	869	4938-52-7
Ethanol, 2-(2-ethoxyethoxy)-	C6H14O3	1012	111-90-0
5-methyl-3-Hexanol	C7H16O	815	623-55-2
1-Undecanol	C11H24O	1357	112-42-5
4-Ethylcyclohexanol	C8H16O	1068	4534-74-1
4-Allyl-1,6-heptadiene-4-ol	C10H16O	1077	10202-75-2
2-Nonanol	C9H20O	1078	628-99-9
(E)-3-Nonen-2-ol	C9H18O	1086	38285-42-6
Allyl mercaptan	C3H6S	613	870-23-5
Cyclohexa-2,4-dienylmethanol	C7H10O	988	154916-94-6
2-methyl-1-Propanol	C4H10O	624	78-83-1
3,4-Dimethylbenzyl Alcohol	C9H12O	1263	6966-10-5
Ester (23)			
Methyl valerate	C6H12O2	785	624-24-8
4-Hexen-1-ol, 5-methyl-2-(1-methylethenyl)-, acetate	C12H20O2	1270	25905-14-0
Propanoic acid, 2-methyl-, propyl ester	C7H14O2	842	644-49-5

Tetrahydrofurfuryl propionate	C8H14O3	1115	637-65-0
2(5H)-Furanone, 3-hydroxy-4,5-dimethyl-	C6H8O3	1088	28664-35-9
Benzyl isocyanate	C8H7NO	1131	3173-56-6
n-Propyl acrylate	C6H10O2	775	925-60-0
Formic acid butyl ester	C5H10O2	783	592-84-7
2-Propanol, 1-(dimethylamino)-, acetate (ester)	C7H15NO2	869	32188-28-6
Benzoic acid, 2-(methylamino)-, methyl ester	C9H11NO2	1372	85-91-6
isocyanato-Cyclohexane	C7H11NO	1216	3173-53-3
2-Heptanol, acetate	C9H18O2	1019	5921-82-4
Acetic acid, hexyl ester	C8H16O2	1011	142-92-7
2-(2-butoxyethoxy)-Ethanol,acetate	C10H20O4	1334	124-17-4
Butanoic acid, 3-methyl-, butyl ester	C9H18O2	1019	109-19-3
Acetic acid, pentyl ester	C7H14O2	910	628-63-7
methyl-Carbamic acid,3-methylphenyl ester	C9H11NO2	1372	1129-41-5
2-Butoxyethyl acetate	C8H16O3	1060	112-07-2
5-Methyl-4-hexene-1-yl acetate	C9H16O2	1068	1000426-93-8
Formic acid, heptyl ester	C8H16O2	1081	112-23-2
6-Octen-1-ol, 3,7-dimethyl-, acetate	C12H22O2	1353	150-84-5
(Z)-3-Octenoic acid, methyl ester	C9H16O2	1091	69668-85-5
Isovaleric acid, 3-methylbutyl-2 ester	C10H20O2	990	1000360-64-9
Acids (12)			
2-methyl-Hexanoic acid	C7H14O2	1042	4536-23-6
2-ethyl-Hexanoic Acid	C8H16O2	1109	149-57-5
Benzoic Acid, 4-hydroxy-	C7H6O3	1371	99-96-7
Butanoic Acid	C4H8O2	775	107-92-6
n-Decanoic Acid	C10H20O2	1372	334-48-5
2-Butenoic Acid, 2-methyl-	C5H8O2	860	13201-46-2
Acetic Acid	C2H4O2	610	64-19-7
Heptanoic Acid	C7H14O2	1073	111-14-8
9-Decenoic acid	C10H18O2	1362	14436-32-9
(E)-2-Butenoic acid	C4H6O2	783	107-93-7
4-Methyloctanoic acid	C9H18O2	1208	54947-74-9
Benzeneacetic acid	C8H8O2	1249	103-82-2
Hydrocarbons (62)			
Nonane, 2,2,4,4,6,8,8-heptamethyl-	C16H34	1294	4390-04-9
Hexane, 2,2,5-trimethyl-	C9H20	767	3522-94-9
trans-1,2-dimethyl-Cyclohexane	C8H16	842	6876-23-9
3-ethyl-Octane	C10H22	965	5881-17-4
4-methylene-1-(1-methylethyl)-Cyclohexene	C10H16	993	99-84-3
Undecane, 2-methyl-	C12H26	1164	7045-71-8
4-Methyl-1,4-heptadiene	C8H14	792	13857-55-1
2-Methyl-2-heptene	C8H16	802	627-97-4
(Z)-3-methyl-2-Pentene	C6H12	603	922-62-3

4,4-dimethyl-1-Pentene	C7H14	623	762-62-9
1-methyl-3-(1-methylethyl)-Cyclohexane	C10H20	976	16580-24-8
propyl-Cyclohexane	C9H18	979	1678-92-8
1-methyl-3-(2-methyl-2-propenyl)-Cyclopentane	C10H18	987	75873-00-6
3,3,6-trimethyl-Decane	C13H28	1165	62338-14-1
3,5-dimethyl-Heptane	C9H20	788	926-82-9
Hexane, 1-methoxy-	C7H16O	793	4747-07-3
1,5-Heptadiene, 2,6-dimethyl-	C9H16	868	6709-39-3
Cyclooctane	C8H16	959	292-64-8
2-methyl-1-Decene	C11H22	1082	13151-27-4
1,9-Decadiene	C10H18	995	1647-16-1
Pentadecane	C15H32	1512	629-62-9
4-methyl-3-Heptene	C8H16	802	4485-16-9
4-methyl-Cyclopentene	C6H10	643	1759-81-5
2,6-Octadiene, 2,6-dimethyl-	C10H18	985	2792-39-4
pentyl-Cyclohexane	C11H22	1178	4292-92-6
trans-1,4-dimethyl-Cyclohexane	C8H16	842	2207-04-7
4-methyl-1-Decene	C11H22	1041	13151-29-6
3-methyl-1-Hexene	C7H14	643	3404-61-3
3-Methylenecyclopentene	C6H8	648	930-26-7
2,3-dimethyl-1,3-Heptadiene	C9H16	868	74779-65-0
(Z)-1,4-Hexadiene	C6H10	616	7318-67-4
1-Decene, 8-methyl-	C11H22	1041	61142-79-8
1-Undecene	C11H22	1091	821-95-4
Tetradecane	C14H30	1413	629-59-4
1,2,4-trimethyl-Cyclopentane	C8H16	783	2815-58-9
Decane, 2,4-dimethyl-	C12H26	1106	2801-84-5
(1-methylethylidene)-Cyclohexane	C9H16	980	5749-72-4
2-methyl-2-Undecene	C12H24	1199	56888-88-1
2,4-dimethyl-2-Pentene	C7H14	638	625-65-0
(E, Z)-2,4-Hexadiene	C6H10	634	5194-50-3
n-Hexane	C6H14	618	110-54-3
2,4,6-trimethyl-Heptane	C10H22	870	2613-61-8
Decane	C10H22	1015	124-18-5
1-(1-methylethyl)-Cyclopentene	C8H14	806	1462-07-3
3,5,5-trimethyl-2-Hexene	C9H18	816	26456-76-8
Tridecane	C13H28	1313	629-50-5
cis-1,3-dimethyl-Cyclohexane	C8H16	842	638-04-0
(Z)-2-Octene	C8H16	824	7642-04-8
3-ethyl-Cyclohexene	C8H14	862	2808-71-1
ethyl-Cyclohexane	C8H16	880	1678-91-7
2,3-dimethyl-Bicyclo [2.2.1] hept-2-ene	C9H14	903	529-16-8
trans,trans-andtrans,cis-1,8-Dimethylspiro[5.5]undecane	C13H24	1356	1000111-73-2

(E)-4,4-dimethyl-2-Pentene	C7H14	641	690-08-4
4,4-dimethyl-2-Pentyne	C7H12	650	999-78-0
5-methyl-1,3-Cyclopentadiene	C6H8	625	96-38-8
1-methoxy-3-methyl-Butane	C6H14O	629	626-91-5
3-(2-methylpropyl)-Cyclohexene	C10H18	997	4104-56-7
1,11-Dodecadiyne	C12H18	1210	20521-44-2
Nonane, 5-methyl-5-propyl-	C13H28	1229	17312-75-3
cis-4,5-Epoxy-(E)-2-decenal	C10H16O2	1264	1000360-26-2
2-methyl-1,6-Heptadiene	C8H14	774	13643-06-6
3-methyl-Heptane	C8H18	773	589-81-1
Aromatics (10)			
2,4-Dimethylstyrene	C10H12	1110	2234-20-0
1-octenyl-Benzene	C14H20	1497	29518-72-7
chloro-Benzene	C6H5Cl	860	108-90-7
Benzene, 1-ethyl-3-methyl-	C9H12	1006	620-14-4
1-ethyl-2,4-dimethyl-Benzene	C10H14	1119	874-41-9
Indane	C9H10	1047	496-11-7
Benzene, 1,2,4,5-tetramethyl-	C10H14	1133	95-93-2
Azulene	C10H8	1069	275-51-4
Benzene, 1-ethyl-2-methyl-	C9H12	1006	611-14-3
2,4-dimethyl-1-(1-methylethyl)-Benzene	C11H16	1155	4706-89-2
Heterocyclic compounds (31)			
1-Tetrazol-2-ylethanone	C3H4N4O	991	51410-11-8
(3R,6S)-2,2,6-Trimethyl-6-vinyltetrahydro-2H-pyran-3-ol	C10H18O2	1255	39028-58-5
threo-2,5-Dimethyl-2-(2-methyl-2-tetrahydrofuryl) tetrahydrofuran	C11H20O2	1287	1000112-56-1
cis-2-(2-Pentenyl) furan	C9H12O	1048	70424-13-4
Maltol	C6H6O3	1063	118-71-8
5H-5-Methyl-6,7-dihydrocyclopentapyrazine	C8H10N2	1095	23747-48-0
5-methyl-2-(methylthio)-4(1H)-Pyrimidinone	C6H8N2OS	1366	20651-30-3
5-methyl-2(5H)-Furanone	C5H6O2	868	591-11-7
3-Amino-5-pyrazolol	C3H5N3O	1194	6126-22-3
4-Amino-2(1H)-pyridinone	C5H6N2O	1080	38767-72-5
2-methoxy-6-methyl-4H-Pyran-4-one	C7H8O3	1099	4225-42-7
1,3,5-Trioxepane	C4H8O3	806	5981-06-6
Piperidine	C5H11N	865	110-89-4
1,2,5-Trimethylpyrrole	C7H11N	856	930-87-0
Ethanone, 1-(2-furanyl)-	C6H6O2	878	1192-62-7
Furan, 2-methyl-	C5H6O	642	534-22-5
Ethanone, 1-(1H-pyrrol-2-yl)-	C6H7NO	1035	1072-83-9
1-methyl-2-Piperidinone	C6H11NO	1040	931-20-4
4-Picoline, 2-(tert-butylthio)-	C10H15NS	1365	18794-36-0
2(3H)-Furanone, 5-ethenyldihydro-5-methyl-	C7H10O2	1049	1073-11-6

6,8-Nonadien-2-one, 6-methyl-5-(1-methylethylidene)-	C13H20O	1387	60714-16-1
1,4-Dihydro-4-oxopyridazine	C4H4N2O	1122	17417-57-1
3-Aminopyridine	C5H6N2	986	462-08-8
2-Piperidinemethanamine	C6H14N2	1169	22990-77-8
2-Ethylpiperazine	C6H14N2	1171	13961-37-0
Pyrazine, 2-methoxy-3-(2-methylpropyl)-	C9H14N2O	1204	24683-00-9
3,4-dihydro-6-methyl-2H-Pyran	C6H10O	1255	16015-11-5
2-Furancarboxylic acid, 2-dimethylaminoethyl ester	C9H13NO3	1257	1000331-10-1
4-[pyrrolidin-2-one-5-yl]-Butan-2-one	C8H13NO2	1258	117155-74-5
2-Oxo-1-methyl-3-isopropylpyrazine	C8H12N2O	1267	78210-68-1
2-Methoxythiophene	C5H6OS	870	16839-97-7
Amines (12)			
2,3-Dimethylcyclohexylamine	C8H17N	1046	42195-92-6
N-Ethyl-2-methylallylamine	C6H13N	783	18328-90-0
4-Propylcyclohexylamine	C9H19N	1184	102653-37-2
Acetamide	C2H5NO	629	60-35-5
Methenamine	C6H12N4	1088	100-97-0
N, N-dimethyl-Urea	C3H8N2O	778	598-94-7
Bicyclo[2.2.2]octan-1-amine	C8H15N	1079	1193-42-6
Cyclobutylamine	C4H9N	684	2516-34-9
(3a. alpha., 6a. alpha., 9a. alpha., 9b. beta.)-Perhydrophenalene	C13H22	1363	40250-64-4
N-ethyl-Cyclopentanamine	C7H15N	959	45592-46-9
Aniline	C6H7N	992	62-53-3
N, N-Dimethylacetamide	C4H9NO	620	127-19-5
Nitrogen compounds (4)			
nitro-Cyclohexane	C6H11NO2	1063	1122-60-7
Isoamyl cyanide	C6H11N	799	542-54-1
2-methyl-Pentanedinitrile	C6H8N2	1044	4553-62-2
Maleic hydrazide	C4H4N2O2	1028	123-33-1
Others (16)			
2H-Pyran,3,6-dihydro-4-methyl-2-(2-methyl-1-propenyl)-	C10H16O	1125	1786-08-9
(1.alpha.,4a.beta.,8a. alpha.)-1,2,3,4,4a,5,6,8a- octahydro-7- methyl-4-methylene-1-(1-methylethyl)-Naphthalene	C15H24	1435	39029-41-9
.beta.-Ocimene	C10H16	976	13877-91-3
2-Methylthiolane, S, S-dioxide	C5H10O2S	1248	1003-46-9
Phenol, 2-(1-methylpropyl)-	C10H14O	1248	89-72-5
2,5-Diethylphenol	C10H14O	1326	876-20-0
isothiocyanato-Cyclohexane	C7H11NS	1251	1122-82-3
2-methoxy-Phenol	C7H8O2	1090	90-05-1
Disulfide, methyl (methylthio)methyl	C3H8S3	1072	42474-44-2
Sarcosine anhydride	C6H10N2O2	1361	5076-82-4
.beta.-Phellandrene	C10H16	964	555-10-2
Diallyl disulphide	C6H10S2	1099	2179-57-9

1-(methylthio)-Butane	C5H12S	769	628-29-5
.alpha.-Muurolene	C15H24	1440	10208-80-7
Pulegone	C10H16O	1212	89-82-7
Propane, 2-chloro-2-nitro-	C3H6ClNO2	774	594-71-8

RI = retention indices calculated. Substance CAS number, Agilent database number.

Table S5. Relative concentrations of volatile compounds of the *T. ovatus* muscle fed different diets (μg/g).

Volatile compounds	D1	D2	D3
Aldehydes (24)	31.17±0.74[a]	27.74±0.62[b]	29.07±1.3[ab]
Ketones (23)	52.61±1.43	52.33±3.27	48.5±2.95
Alcohols (28)	60.22±1.36[a]	51.17±1.04[b]	51.63±1.72[b]
Esters (23)	17.13±1.91	13.23±1	13.24±1.64
Acids (12)	12.37±1.61	9.74±0.68	10.25±1.47
Hydrocarbons (62)	206.89±6.85[a]	177.63±5.35[b]	195.89±10[ab]
Aromatics (10)	22.16±0.75	23.06±1.41	20.73±1.45
Heterocyclic compounds (31)	151.81±4.68	162.45±11.79	148.83±11.2
Amines (12)	12.96±0.45[a]	10.05±0.31[b]	10.62±0.53[b]
Nitrogen compounds (4)	3.77±0.15	3.22±0.12	3.31±0.27
Others (20)	1.86±0.09	2.00±0.14	1.89±0.15
Total (245)	572.94±15.61	535.61±15.99	533.95±20.8

Results are presented as the mean ± SEM (n = 3). Within each row, means without sharing a common letter are significantly different ($p < 0.05$).

Table S6. Contents of differential volatile compounds (VIP > 1) in the muscle of *T. ovatus* fed different diets (μg/g).

Volatile compounds	Dietary groups			VIP
	D1	D2	D3	
1-Decene, 8-methyl-	0.09±0.01[a]	0.05±0[b]	0.05±0[b]	1.81
Formic acid, heptyl ester	0.12±0.01[a]	0.05±0[b]	0.04±0[b]	1.80
Ethanone, 1-(1H-pyrrol-2-yl)-	0.45±0.02[a]	0.3±0.03[b]	0.23±0.01[b]	1.76
Bicyclo[2.2.2]octan-1-amine	0.07±0.01[a]	0.02±0[b]	0.02±0[b]	1.74
1-Undecanol	0.06±0[a]	0.02±0[b]	0.01±0[b]	1.73
6-Octen-1-ol, 3,7-dimethyl-, acetate	0.06±0.01[a]	0.02±0[b]	0.02±0[b]	1.72
Benzyl methyl ketone	0.02±0[b]	0.02±0[b]	0.05±0.01[a]	1.71
Maleic hydrazide	2.82±0.11[a]	2.08±0.09[b]	1.74±0.05[b]	1.69
2-methyl-Pentanedinitrile	0.19±0.01[a]	0.14±0.01[b]	0.12±0[b]	1.67
3,3-dimethyl-2-Hexanone	0.03±0[b]	0.04±0[b]	0.05±0[a]	1.67
2,4,6-trimethyl-Heptane	0.03±0[b]	0.04±0[a]	0.05±0[a]	1.65
Decane	0.05±0[a]	0.03±0[b]	0.03±0[b]	1.64
4-methyl-1-Decene	0.14±0.01[a]	0.07±0[b]	0.08±0.01[b]	1.63
3-(2-methylpropyl)-Cyclohexene	0.04±0[b]	0.03±0[b]	0.07±0[a]	1.63
1,2,5-Trimethylpyrrole	0.18±0.01[b]	0.2±0[ab]	0.23±0.01[a]	1.62
1,11-Dodecadiyne	0.2±0.03[a]	0.12±0.01[b]	0.09±0.01[b]	1.60
Hexane, 2,2,5-trimethyl-	0.4±0.02[a]	0.09±0.01[b]	0.1±0.01[b]	1.59
BenzeneacetAldehyde	0.22±0.01[a]	0.05±0.01[b]	0.06±0.01[b]	1.59
1-methyl-2-Piperidinone	0.04±0	0.03±0	0.03±0	1.58
2,3-Dimethylcyclohexylamine	0.23±0.01[a]	0.17±0.01[b]	0.14±0.01[c]	1.57
Butanoic acid, 3-methyl-, butyl ester	0±0	0±0	0.01±0	1.56
2,6-Octadiene, 2,6-dimethyl-	0.12±0.01[a]	0.05±0.01[b]	0.05±0.01[b]	1.55
1-methyl-3-(1-methylethyl)-Cyclohexane	0.15±0.01[a]	0.06±0.01[b]	0.06±0.01[b]	1.54
4-Propylcyclohexylamine	0.9±0.16[a]	0.57±0.08[ab]	0.29±0.04[b]	1.54
4-Hexen-1-ol, (E)-	0.05±0	0.05±0	0.06±0	1.54
3,3,6-trimethyl-Decane	1.21±0.19[a]	0.76±0.12[ab]	0.43±0.06[b]	1.53
2,6-Octadienal, 3,7-dimethyl-, (E)-	0.5±0.09[a]	0.32±0.05[b]	0.17±0.02[b]	1.53
Undecane, 2-methyl-	1.12±0.18[a]	0.73±0.12[ab]	0.39±0.05[a]	1.53
2-Nonen-1-ol	1.63±0.27[a]	1.04±0.16[ab]	0.56±0.08[b]	1.52
2-Piperidinemethanamine	0.64±0.11[a]	0.39±0.05[ab]	0.21±0.04[b]	1.52

1-Hepten-3-ol	0.05±0	0.05±0	0.06±0	1.52
Acetic acid, hexyl ester	0.05±0[a]	0.03±0[b]	0.03±0[b]	1.52
2,3-dimethyl-1,3-Heptadiene	0.08±0	0.09±0	0.1±0.01	1.52
2-Ethylpiperazine	0.96±0.18[a]	0.61±0.1[b]	0.31±0.05[b]	1.50
4-Methyl-1,4-heptadiene	0.02±0	0.01±0	0.01±0	1.50
Pentadecane	1.12±0.22[a]	0.36±0.02[b]	0.4±0.04[b]	1.48
Tetradecane	0.16±0.03[a]	0.08±0[b]	0.08±0.01[b]	1.48
Ethanone, 1-(2-furanyl)-	0.05±0[b]	0.07±0[a]	0.07±0[a]	1.48
1,6-Heptadien-4-ol	0.06±0[b]	0.08±0[a]	0.08±0[a]	1.46
2-Butoxyethyl acetate	0.01±0	0.02±0	0.02±0	1.46
2,5-Dimethylcyclohexanone	0.04±0[a]	0.02±0[b]	0.02±0[b]	1.45
1-methyl-3-(2-methyl-2-propenyl)-Cyclopentane	0.34±0.02[a]	0.26±0.01[b]	0.23±0.01[b]	1.45
1-Dodecanethiol	0.22±0.04[a]	0.07±0.01[b]	0.08±0.01[b]	1.43
2-methyl-Hexanoic acid	0.21±0.01[a]	0.17±0.01[ab]	0.13±0[b]	1.43
Cyclobutylamine	3.25±0.15[a]	1.74±0.09[b]	1.99±0.08[b]	1.43
3-ethyl-Cyclohexene	0.11±0[b]	0.13±0[a]	0.14±0.01[a]	1.43
Piperidine	0.95±0.06	1.11±0.05	1.15±0.03	1.43
1-methoxy-3-methyl-Butane	0.3±0.01	0.25±0.01	0.29±0.02	1.42
Citral	0.95±0.16[a]	0.64±0.12[b]	0.33±0.07[b]	1.42
2,3-Butanediol, 2,3-dimethyl-	1.11±0.13[a]	0.44±0.1[b]	0.46±0.09[b]	1.42
2-Octen-1-ol, (E)-	0.02±0	0.02±0	0.02±0	1.41
Cyclohexa-2,4-dienylmethanol	0.09±0.01[a]	0.07±0.01[ab]	0.06±0[b]	1.41
2-Methoxythiophene	5.89±0.35[b]	6.7±0.13[ab]	6.94±0.17[a]	1.41
2(3H)-Furanone, 5-ethenyldihydro-5-methyl-	3.29±0.12[a]	2.67±0.25[b]	2.07±0.01[b]	1.41
2-Propanol, 1-(dimethylamino)-, acetate (ester)	0.23±0.01	0.26±0.01	0.27±0.01	1.41
1-Undecene	0.8±0.06[a]	0.24±0.02[b]	0.27±0.02[b]	1.41
1-Hexanol	0.58±0.04	0.68±0.03	0.7±0.02	1.41
chloro-Benzene	0.36±0.02	0.41±0.02	0.43±0.01	1.40
(E)-3-Nonen-2-ol	1.78±0.12[a]	0.51±0.02[b]	0.6±0.03[b]	1.39
2,3-dimethyl-Bicyclo[2.2.1]hept-2-ene	0.03±0[a]	0.01±0[b]	0.01±0[b]	1.38
pentyl-Cyclohexane	0.32±0.05[a]	0.35±0.02[a]	0.14±0.02[b]	1.37
2,3,4-Trimethyl-1-pentanol	1.23±0.09	1.32±0.07	1.39±0.03	1.37

1,5-Heptadiene, 2,6-dimethyl-	0.05±0[b]	0.06±0[ab]	0.07±0[a]	1.36
2-methyl-1-Decene	0.04±0[a]	0.02±0.01[b]	0.02±0[b]	1.32
ethyl-Cyclohexane	0.21±0.01[b]	0.26±0.01[a]	0.25±0[a]	1.32
5-methyl-2(5H)-Furanone	0.11±0.01	0.13±0.01	0.13±0	1.32
1-Tetrazol-2-ylethanone	0.93±0.08	1.06±0.06	1.17±0.13	1.32
Benzoic acid, 2-(methylamino)-, methyl ester	13.96±2.82[a]	10.15±1.13[a]	6.78±0.42[b]	1.31
Ethanone, 1-(2-hydroxy-5-methylphenyl)-	1.33±0.26	0.97±0.09	0.7±0.03	1.28
n-Propyl acrylate	0.03±0	0.04±0	0.04±0	1.28
2-methyl-3-Hexanone	0.1±0.02[a]	0.05±0.01[ab]	0.05±0.01[b]	1.28
3-methyl-Hexanal	0.71±0.08[a]	0.39±0.12[ab]	0.28±0.07[b]	1.28
2,2-dimethyl-Cyclopentanone	0.03±0[a]	0.01±0[b]	0.01±0[b]	1.27
(3a.alpha., 6a.alpha., 9a.alpha., 9b.beta.)-Perhydrophenalene	0.75±0.14	0.54±0.05	0.4±0.02	1.25
Pulegone	0.04±0.01	0.04±0	0.02±0	1.25
3-methyl-Cyclopentanone	0.08±0.01[a]	0.04±0.01[ab]	0.04±0.01[b]	1.21
cis-2-(2-Pentenyl)furan	0.46±0.02[a]	0.36±0.01[b]	0.32±0.02[b]	1.21
methyl-Carbamic acid,3-methylphenyl ester	0.43±0.08	0.33±0.04	0.23±0	1.21
2,4-Octadienal, (E,E)-	0.48±0.01[a]	0.18±0[b]	0.15±0.03[b]	1.20
(E)-4-Heptenal	0.04±0[a]	0.02±0[b]	0.02±0[b]	1.19
2,5-Diethylphenol	0.01±0	0.01±0	0.01±0	1.19
9-Decenoic acid	3.24±0.62	2.45±0.25	1.77±0.09	1.18
N,N-dimethyl-Urea	0.77±0[b]	0.84±0.01[a]	0.82±0.01[a]	1.18
Propanoic acid, 2-methyl-, propyl ester	0.51±0.06	0.27±0.08	0.23±0.06	1.18
Heptanal	0.1±0[a]	0.06±0[b]	0.06±0.01[b]	1.18
2-Methyl-2-heptene	0.13±0[a]	0.09±0.01[b]	0.1±0.01[b]	1.18
4-methyl-3-Penten-1-ol	1.29±0.12	0.69±0.21	0.57±0.16	1.17
6,8-Nonadien-2-one, 6-methyl-5-(1-methylethylidene)-	1.16±0.23	0.87±0.08	0.65±0.02	1.16
Formic acid butyl ester	0.14±0	0.14±0	0.14±0	1.15
3-methyl-Heptane	1.17±0.01	1.18±0.03	1.18±0.01	1.15
cis-1,3-dimethyl-Cyclohexane	1.02±0.11	0.56±0.18	0.45±0.12	1.15
3-Hexanone-2,2,4,4-d4	2.5±0	2.5±0	2.5±0	1.14
1,9-Decadiene	0.09±0.01	0.09±0.01	0.1±0.01	1.14

2-methyl-Cyclopentanone	2.3±0.29	1.25±0.39	1.01±0.29	1.14
2,3-Hexanedione	1.2±0.01	1.19±0.04	1.2±0.01	1.14
2-Undecenal	0.03±0[a]	0.01±0[b]	0.02±0[b]	1.13
Benzoic Acid, 4-hydroxy-	9.09±1.82[a]	6.83±0.62[ab]	5.16±0.1[b]	1.13
n-Decanoic Acid	0.12±0.02	0.09±0.01	0.06±0	1.12
Tridecane	0.14±0[a]	0.06±0[b]	0.08±0.01[b]	1.11
N-Ethyl-2-methylallylamine	0.16±0.01	0.18±0	0.17±0.01	1.10
2-methyl-1,6-Heptadiene	0.27±0	0.27±0.01	0.27±0	1.10
Hexane, 1-methoxy-	6.09±0.02	6.07±0.14	6.01±0.06	1.09
Methyl valerate	0.35±0	0.37±0.01	0.36±0	1.09
2-Butenoic acid, 2-methyl-	0.17±0.01	0.17±0.01	0.17±0.01	1.09
Butanoic Acid	0.12±0	0.12±0	0.12±0	1.07
Sarcosine anhydride	0.1±0.02	0.09±0.01	0.06±0.01	1.05
ParAldehyde	0.01±0	0.01±0	0.01±0	1.04
Acetic acid, pentyl ester	0.04±0[a]	0.03±0[b]	0.03±0[b]	1.03

Table S7. Concentrations and odor activity values (data in brackets) of differential volatiles in the muscle of *T. ovatus* fed different diets.

Volatile compounds	Dietary groups						Threshold[a] (μg/kg)	Flavor description
	D1		D2		D3			
3,4,5-trimethyl-2-Cyclopenten-1-one	5.02[a]	(5.02)	4.56[ab]	(4.56)	4.18[b]	(4.18)	1	Sweet, fruity
2(3H)-Furanone, 5-ethenyldihydro- 5-methyl	3.29[a]	(1.10)	2.67[b]	(0.89)	2.07[b]	(0.69)	3	Caramel, maple
2-methyl-undecane	1.12[a]	(0.86)	0.73[ab]	(0.56)	0.39[b]	(0.30)	1.3	Mild oily
Benzoic acid, 2-(methylamino)-, methyl ester	13.96[a]	(0.69)	10.15[a]	(0.50)	6.78[b]	(0.33)	20.3	Sweet, apple
Citral	1.08[a]	(0.25)	0.63[b]	(0.15)	0.43[b]	(0.10)	4.3	Lemon, orange
3,3,6-trimethyl-Decane	1.21[a]	(0.12)	0.76[ab]	(0.08)	0.43[b]	(0.04)	10	Alkane-like
3-methyl-2-Butanol	41.03[a]	(0.034)	33.96[b]	(0.028)	34.78[b]	(0.028)	1200	Malty, rancid, pungent
2-methyl-Cyclopentanone	2.19[a]	(0.02)	1.12[b]	(0.01)	1.22[b]	(0.01)	100	Solventy
propyl-Cyclohexane	3.09[a]	(0.017)	2.46[b]	(0.013)	2.55[b]	(0.014)	181	Sweet
Benzoic Acid, 4-hydroxy-	9.09[a]	(0.01)	6.83[b]	(<0.01)	5.16[b]	(<0.01)	745.2	Floral
2,3-Butanediol, 2,3-dimethyl	1.11[a]	(0.01)	0.44[b]	(<0.01)	0.46[b]	(<0.01)	95.1	Pungent, buttery
(1-methylethylidene)-Cyclohexane	3.2[a]	(0.002)	2.55[b]	(0.001)	2.61[b]	(0.001)	1310	Minty
2-Methoxythiophene	5.88[b]	(<0.01)	6.57[a]	(<0.01)	6.8[a]	(<0.01)	3000	Slightly sweet
9-Decenoic acid	3.24[a]	(<0.01)	2.45[b]	(<0.001)	1.77[b]	(<0.001)	3000	Rancid, sour, fatty
4-methyl-3-Penten-1-ol	1.29[a]	(<0.01)	0.69[b]	(<0.01)	0.57[b]	(<0.01)	358.1	Mushroom, grassy
2-Nonen-1-ol	1.63[a]	(<0.01)	1.04[ab]	(<0.01)	0.56[b]	(<0.001)	1000	Sweet, floral
(E)-3-Nonen-2-ol	1.78[a]	(<0.001)	0.51[b]	(<0.001)	0.6[b]	(<0.001)	70000	Greeen, cucumber
Pentadecane	1.12[a]	(<0.001)	0.36[b]	(<0.001)	0.4[b]	(<0.001)	1800	Alkane-like
2-methyl-3-Pentanone	1.75[a]	(<0.001)	0.61[b]	(<0.001)	1.25[ab]	(<0.001)	4500	Sweet, fruity
Acetamide	1.72[a]	(<0.001)	1.48[b]	(<0.001)	1.67[ab]	(<0.001)	20000	Amine, burnt, oily
2,4-dimethyl-2-Pentene	126.32[a]		106.95[b]		120.72[ab]		nf	Sweet and fruity
N-ethyl-Cyclopentanamine	4.8[a]		3.81[b]		3.94[b]		nf	Fishy, ammonia
Cyclobutylamine	3.25[a]		1.74[b]		1.99[b]		nf	Ammonia, fishy

1,3,5-Trioxepane	2.85[a]	2.59[b]	2.53[b]	nf	Slightly sweet
Cyclooctane	2.32[a]	2.1[ab]	1.91[b]	nf	Slightly sweet

[a] The threshold value of volatile used in the present study was obtained from the reported literature (Ruth, 1986; Giri et al., 2010; Murnane et al., 2013; Guo et al., 2021; Chen et al., 2017; Zhang et al., 2020; Yin et al., 2021; Zhang et al., 2021; Yang et al., 2022; Yan et al., 2024)
